# The relationship between resilience and personality traits in doctors: implications for enhancing well being

**DOI:** 10.7717/peerj.216

**Published:** 2013-11-19

**Authors:** Diann S. Eley, C. Robert Cloninger, Lucie Walters, Caroline Laurence, Robyn Synnott, David Wilkinson

**Affiliations:** 1 School of Medicine, The University of Queensland, Queensland, Australia; 2 Department of Psychiatry and Sansone Centre for Well-Being, Washington University, USA; 3 Rural Clinical School, Flinders University, South Australia, Australia; 4 Discipline of General Practice, School of Population Health and Clinical Practice, University of Adelaide, South Australia, Australia; 5 Corporate Engagement & Advancement, Macquarie University, NSW, Australia

**Keywords:** Temperament, Resilience, Character, Well being, Doctors

## Abstract

**Objective.** The health and well being of medical doctors is vital to their longevity and safe practice. The concept of resilience is recognised as a key component of well being and is an important factor in medical training to help doctors learn to cope with challenge, stress, and adversity. This study examined the relationship of resilience to personality traits and resilience in doctors in order to identify the key traits that promote or impair resilience.

**Methods.** A cross sectional cohort of 479 family practitioners in practice across Australia was studied. The Temperament and Character Inventory measured levels of the seven basic dimensions of personality and the Resilience Scale provided an overall measure of resilience. The associations between resilience and personality were examined by Pearson product-moment correlation coefficients, controlling for age and gender (α = 0.05 with an accompanying 95% confidence level) and multiple regression analyses.

**Results.** Strong to medium positive correlations were found between Resilience and Self-directedness (*r* = .614, *p* < .01), Persistence (*r* = .498, *p* < .01), and Cooperativeness (*r* = .363, *p* < .01) and negative with Harm Avoidance (*r* = .−555, *p* < .01). Individual differences in personality explained 39% of the variance in resilience [*F*(7, 460) = 38.40, *p* < .001]. The three traits which contributed significantly to this variance were Self-directedness (β = .33, *p* < .001), Persistence (β = .22, *p* < .001) and Harm Avoidance (β = .19, *p* < .001).

**Conclusion.** Resilience was associated with a personality trait pattern that is mature, responsible, optimistic, persevering, and cooperative. Findings support the inclusion of resilience as a component of optimal functioning and well being in doctors. Strategies for enhancing resilience should consider the key traits that drive or impair it.

## Introduction

The well-being of physicians is crucial for their professional effectiveness as well as for the resilience of their own health and happiness. The failure or loss of resilience in physicians leads to burn-out, which is a major concern in medical centres because of its impact on health care (Shanafelt, Sloan & Habermann, 2003; Riley, 2004; Howe, Smajdor & Stockl, 2005; Eckleberry-Hunt et al., 2009). The literature on personality traits of doctors is substantial, and dominant traits and trait patterns of students, doctors and specific specialities have been investigated primarily for their relevance to psychology and education research in gaining a better understanding of career preferences (Borges & Savickas, 2002; Borges & Gibson, 2005; Eley, Young & Przybeck, 2009a), academic progression (Ferguson, James & Madeley, 2002; Lievens et al., 2002; Hojat et al., 2003), clinical skills (Hojat, Callahan & Gonnella, 2004; Manuel, Borges & Gerzina, 2005), and professional behaviour (Munro, Bore & Powis, 2005; Hodgson et al., 2007).

Resilience stands out as a key component of well being in the literature on the determinants of health (Dyrbye et al., 2005; Cloninger & Zohar, 2011; Drybye & Shanafelt, 2012), and healthy coping with trauma (Gil & Caspi, 2006; North, Abbacchi & Cloninger, 2012; North & Cloninger, 2012). The nursing literature also stresses the development of resilience as a coping mechanism for workforce issues (Jackson, Firtko & Edenborough, 2007; McAllister & McKinnon, 2009). However there is little in the medical education literature that measures levels of resilience in students or doctors (Howe, Smajdor & Stockl, 2005). As a result, it is uncertain what needs to be done to develop and support resilience in the providers of medical care, although some work has been carried out to treat burn-out (Krasner et al., 2009).

Resilience can be considered as a process of adaptation to adversity and stress. Resilient individuals tend to recover from setbacks or trauma and portray a common set of characteristics that help them cope with challenges in life (McAllister & McKinnon, 2009; Herrman et al., 2011). The fields of psychiatry, psychology, and psychotherapy have investigated relationships between resilience and personality dimensions to understand what drives healthy coping styles and adaptive behaviours. Research with various instruments such as the NEO Five Factor Inventory (Costa & McCrae, 1992) and the Temperament and Character Inventory (TCI) (Cloninger & Zohar, 2011) have shown resilience to be associated with coping with prior trauma, and health-promoting personality traits in adults (Campbell-Sills, Cohan & Stein, 2005; Simeon et al., 2007), particularly the pre-exposure temperament trait of low Harm Avoidance (Gil & Caspi, 2006), and the character trait of high Self-directedness (North, Abbacchi & Cloninger, 2012; North & Cloninger, 2012).

Medical doctors and students benefit from a high degree of resilience which helps them cope with the obvious challenges of their profession, such as high workload, emotional and physical demands and expectations (Howe, Smajdor & Stockl, 2005; Drybye & Shanafelt, 2012). Doctors need to constantly respond to challenges in their practice, and resilient individuals are better equipped to meet these challenges, learn from them, and to continue to cope with the increasing workloads and stressful situations of medical practice. The temperament trait of high Persistence in synergy with high Self-directedness and low Harm Avoidance is often beneficial in successful adaptation to such demanding work situations (Cloninger et al., 2012).

The medical education literature, especially over the past decade, has reported the prevalence of burnout, depression and distress in students, (Dahlin, Joneborg & Runeson, 2005; Dyrbye & Shanafelt, 2006; Dyrbye et al., 2008) and doctors, (Williams et al., 2002; West et al., 2009) and the connection between doctors’ well being and safe practice, (West et al., 2009) and professionalism (Tempski, Martins & Paro, 2006).

Resilience is considered to be a dynamic ‘process’ that manifests itself in response to life circumstances and individual personality profiles, and is a marker of well being and a psychologically mature personality (Richardson, 2002; Tempski, Martins & Paro, 2006; Cloninger & Zohar, 2011; Drybye & Shanafelt, 2012). Psychological maturity is demonstrated by a character profile that has high levels of Self-directedness and Cooperativeness (Cloninger, Svrakic & Pryzbeck, 1993; Svrakic & Whitehead, 1993), which is often bolstered by temperament traits of low Harm Avoidance and high Persistence (Cloninger et al., 2012). In its complete sense, psychological maturity is a strong predictor of someone being able to cope with life’s challenges and to bounce back from adversity, or in other words, being resilient (Cloninger, Salloum & Mezzich, 2012).

The search for appropriate ways to assess resilience as an individual trait has been prominent in personality research since the 1950s. However, when resilience has been considered along with measures of personality, some studies fail to find that resilience adds any information beyond what is measured by standard personality tests like the Five Factor Model (Waaktaar & Torgersen, 2010), while other studies do find that resilience adds information to personality (Friborg et al., 2005). Therefore, we seek in this paper to see how well personality is able to account for resilience and measures of its components.

This study posits that resilience is a process that is influenced by one’s combination of personality traits and their environment. Because the environment is rarely stable it follows that behaviour results from a dynamic process of responding to and coping with life challenges (Cloninger, Salloum & Mezzich, 2012; Josefsson et al., 2013). Interdisciplinary studies support the view that genetics and the environment contribute to an individual’s capacity for resilience (Feder, Nestler & Charney, 2009). We investigated the relationship between the pattern of personality traits and measures of resilience in a sample of FPs. We hypothesised that resilience would reveal meaningful relationships with a pattern of personality traits and expand our understanding of what contributes to a resilient personality and overall well being in doctors.

## Materials and Methods

In 2011 a cross sectional cohort design sampled FPs across all states in Australia. Access was via Regional Family Practitioner Training Providers and the Australian College of Rural and Remote Medicine.

### Ethics

Ethics approval was obtained through the National Ethics Application Form of the National Health and Medical Research Council of Australia and subsequent approval from The University of Queensland [#2010001618], the University of Adelaide [#H-047-2011] and Flinders University [#5134].

### Measures

A self-report questionnaire included the Temperament and Character Inventory (TCI-R140) (Cloninger et al., 1994) to identify seven basic dimensions of personality and the Resilience Scale (Wagnild, 2009) which measures the essential characteristics of resilience. Because personality trait levels are known to vary by sex and age, these variables were included in the analysis (Cloninger, 2004; Eley, Young & Prysbeck, 2009b). Questionnaire administration was by a one-time invitation (no reminders) using identical versions either hard copy, administered in a classroom situation, or on-line (Survey Monkey©).

### Temperament and character

The TCI is based on Cloninger’s psychobiological theory of personality which distinguishes between the personality domains of moderately stable temperament traits that vary according to individual differences in behavioural conditioning [i.e., the emotional core of personality] and character traits that develop across the lifespan toward socially approved norms [i.e., the cognitive domain of personality] (Cloninger, 2004; Josefsson et al., 2013). The TCI is validated in adult populations across the world including the USA, Australia, Europe, Israel and Asia and each scale correlates with other tests of personality, such as the five-factor personality model, performing as well or better than other modern tests in predicting mature coping (Picardi et al., 2005; Grucza & Goldberg, 2007). We administered the 140 item version using a five point Likert scale from 1 (absolutely false) to 5 (absolutely true). The four temperament traits are Novelty Seeking, Harm Avoidance, Reward Dependence and, Persistence. The three character traits are Self-directedness, Cooperativeness and Self Transcendence. Each trait is multifaceted. High and low descriptors are summarized in Table 1.

**Table 1:** Table of trait descriptors. Temperament and character trait descriptors. (Adapted from Cloninger et al., 1994.)

Temperament traits	*Represents*.........	LOW SCORES	*to*	HIGH SCORES
Novelty Seeking	*Exploratory activity in response to novelty*	*Orderly, reflective, tolerant, reserved*	↔	*Exploratory, curious, seeks challenge*
Harm Avoidance	*Worry in anticipation of problems*	*Confident, accepting of uncertainty & risk*	↔	*Worrying, anxious, unable to accept risk*
Reward Dependence	*Dependence on approval of others*	*Not influenced by others, objective, insensitive*	↔	*Needs to please, warm, attached*
Persistence	*Industriousness of behaviour despite obstacles*	*Quitting, underachiever, erratic, un-ambitious*	↔	*Ambitious, diligent, perfectionist*
**Character traits**	***Represents***.........	**LOW SCORES**	***to***	**HIGH SCORES**
Self-directedness	*Responsibility, goal oriented & self-confidence*	*Blaming, ineffective, unreliable, unreliable*	↔	*Conscientious, self accepted, reliable,*
Cooperativeness	*Tolerance, cooperativeness & empathy*	*Intolerant, unhelpful, opportunistic, critical*	↔	*Tolerant, agreeable, constructive, empathic*
Self Transcendence	*View of self in relation to the universe as a whole*	*Impatient, proud, materialistic, practical*	↔	*Patient, humble, spiritual, creative*

### Resilience

The Resilience Scale is a self report measure of an individual’s ability to respond to adversity. The 26 item version uses a 7 point Likert-scale from Strongly Disagree (1) to Strongly Agree (7). The scale reflects five core characteristics of resilience: perseverance, equanimity, meaningfulness, self reliance and existential aloneness (Wagnild, 2009). Perseverance indicates a willingness to persist despite adversity. Equanimity refers to balance – the ability to ‘take what comes’ in life. Meaningfulness is the acknowledgment that life has a purpose and is therefore worth living. Self reliance reflects an individual’s self belief and their dependence on their own strengths and past success to support their decisions. Existential aloneness is the awareness that every person is unique and this realisation allows a sense of independence and freedom. Our analysis used the single composite score of resilience as its primary planned criterion of resilience but also explored relations with the subscales to clarify understanding.

### Analysis

Tests of normality (Kolmogorov–Smirnov statistic) showed the TCI and Resilience scores for the whole sample were normally distributed. The internal consistency (Cronbach alpha) of the Resilience Scale was .89, the TCI ranged from .84 to .88 for the character and from .76 to .89 for the temperament scales. Chi-square tests examined proportions in the demographic variables. Two-way ANOVA with post-hoc pair-wise comparisons examined differences between traits by sex and age. The relationship between measures of temperament and character dimensions and resilience was investigated by Pearson product-moment correlation coefficients (two tailed) controlling for age and sex. Standard multiple regression analysis was used to determine the amount of variance in Resilience scores is explained by temperament and character traits. All tests used α = 0.05 with an accompanying 95% confidence level and analysed using SPSS 21 (SPSS Inc, Chicago, IL, USA).

## Results

### Demographic data

The response rate was 61%: 479 out of the 785 FP trainees identified completed our questionnaire. The majority (*n* = 287; 59%) was female and aged between 22 and 31 years (*n* = 225 of 479; 47%). Another 34% (*n* = 161 of 479) were between 32 and 41 years. The spread in ages is representative of Australian medical programs which range in duration from 4 to 6 years. Additionally entry into vocational training can occur any time after completion of intern training, i.e., post graduate years 1 and 2 with a trend toward undertaking specialist training after practising for a few years in family practice. Over 90% were Australian born, i.e., less than 10% were international medical graduates.

### Levels of personality traits and resilience among the whole sample

As shown in Table 2, ranking the trait levels of the whole sample with published population norms (Cloninger et al., 1994; Wagnild, 2009) showed our FP trainees to be very high in Reward Dependence, Persistence, Self-directedness and Cooperativeness, average in levels of Novelty Seeking and Harm Avoidance, and low in Self Transcendence. The sample ranked moderately high in Resilience.

**Table 2:** Table of trait scores. Raw mean scores and standard deviations of levels of temperament, character and resilience ranked against population norms (*N* = 479).

Trait	Mean	Std. deviation	Mean scores ranked with population norms^*^
Novelty Seeking	53.25	7.79	Average
Harm Avoidance	54.65	11.97	Average
Reward Dependence	69.80	9.89	Very high
Persistence	71.34	9.45	Very high
Self directedness	77.08	9.34	Very high
Cooperativeness	80.71	7.95	Very high
Self Transcendence	42.34	10.92	Low
Resilience	143.33	16.42	Moderately high^**^
Self reliance	27.93	4.34	Not available
Meaning	27.76	3.95	”
Equanimity	25.84	4.07	”
Perseverance	28.11	3.89	”
Existential aloneness	27.44	4.96	”

**Notes.**

* TCI Normative Population based on Cloninger et al. (1994). Very low, 0–16.7%; low, 17–33%; average, 34–66.7%; high, 67–83.3%; very high, 84–100%.

** Resilience population norms based on Wagnild (2009). Very low, 25–100; low, 101–115; moderately low, 116–130; moderately high, 131–145; high, 145–160; very high, 161–175.

Females were higher than males in levels (means, standard deviations) of Harm Avoidance (56.62, 11.69 vs 51.69, 11.81: *t* = 4.509, 477; *p* < 0.001), Reward Dependence (72.77, 9.32 vs 65.37, 9.05: *t* = 8.615, 477; *p* < 0.001) and Cooperativeness (81.90, 7.77 vs 78.92, 7.92: *t* = 4.089, 477; *p* < 0.001), and lower in Novelty Seeking (52.59, 7.58 vs 54.23, 8.02: *t* = 2.261, 477; *p* < .02) and the Existential Aloneness scale of Resilience (26.89, 5.00 vs 28.26, 4.88: *t* = 2.986, 477; *p* < .0203). Younger doctors (22–31 years) are higher than all older (32–61) in Reward Dependence (71.34, 10.28 vs 67.58, 9.22: *F* = 5.041, 3; *p* < 0.002.) Although significant, effect sizes for differences in gender and age were small. No other differences were detected within the sample.

### Relationships between resilience and personality traits

The relationships among the measures of temperament and character traits and resilience are detailed in Table 3. Resilience was most strongly correlated with high Self-directedness and low Harm Avoidance. It was moderately correlated with high Persistence and high Cooperativeness. Resilience had no significant correlation with Novelty Seeking, Reward Dependence and Self Transcendence.

**Table 3:** Table of correlation coefficients^*^. Pearson correlation coefficients between temperament and character traits and the Resilience Scale; total score, subscale scores and individual question (‘I am resilient’) (*N* = 479).

	Resilience total score	‘I am Resilient’	Self reliance	Meaning	Equanimity	Perseverance	Existential aloneness	HA	PS	SD	CO
Resilience – total score	1	.654	.856	.780	.810	.800	.843	−.426	.446	.530	.258
‘I am resilient’ single item			.603	.377	.481	.496	.534	−.343	.357	.348	.197
Self reliance				.605	.639	.714	.698	−.383	.419	.419	.246
Meaning					.594	.622	.604	−.285	.311	.437	.241
Equanimity						.562	.684	−.446	.179	.404	.186
Perseverance							.589	−.384	.573	.485	.241
Existential aloneness								−.325	.234	.328	.077
Harm Avoidance (HA)									−.405	−.560	−.144
Persistence (PS)										.457	.274
Self-directedness (SD)											.495
Cooperativeness (CO)											1

**Notes.**

* All correlations are significant at the 0.001 level (2-tailed) *N* = 479.

Strength of correlation; medium: *r* = .30 to .49; strong: *r* = .50 to 1.0.

Considering the relations among the TCI dimensions in this high functioning sample, low Harm Avoidance was most strongly correlated with Self-directedness and moderately with Novelty Seeking, Persistence, and Cooperativeness. Cooperativeness was strongly correlated with Self Directedness, and moderately with Reward Dependence and Persistence. Likewise Self Directedness and Persistence were moderately correlated. Self Transcendence was uncorrelated with the other TCI dimensions.

Preliminary tests for multiple regression analysis showed there were no violations to the assumptions of normality, linearity and multicollinearity. The total variance in resilience explained by the whole forward-selection regression model was 39%, *F* (7, 460) = 38.40, *p* < .001. The three traits which contributed significantly to this variance were Self-directedness (β = .33, *p* < .001), Persistence (β = .22, *p* < .001) and Harm Avoidance (β = −.19, *p* < .001).

## Discussion

This study examined the relationship between measures of resilience and personality in a high functioning sample of physicians. These data provide the first information about the personality correlates of resilience in physicians. Resilience has strong and significant relationships with a pattern of traits that support high functioning in a demanding and stressful profession with a high risk of burn-out (Riley, 2004; Eckleberry-Hunt et al., 2009). Our sample of family practitioners had a psychologically mature and confident personality profile, characterized by high levels of Self-directedness, Cooperativeness, and Persistence, and low levels of Harm Avoidance. This profile corresponds to personality features that distinguish healthy people from those who are unhealthy in samples from the general population in the USA (Cloninger, 2004), Europe (Josefsson et al., 2011) and Asia (Kijma et al., 2000; Cloninger & Zohar, 2011). These findings confirm that resilience is closely related to the more general constructs of health and well-being, as discussed elsewhere (Cloninger, Salloum & Mezzich, 2012). These findings support our recommendation that resilience as a trait should not be considered in isolation but as an expression of interactions among multiple components of personality that can enhance or impair it. It is perhaps appropriate that the effectiveness of physicians as health care providers may be enhanced by their own health and well-being.

A recent study examined this relationship using a different measure of Resilience (Conner-Davison Resilience Scale) in a sample of Korean university students (Kim, Lee & Lee, 2013). There are interesting similarities and differences between these studies. The most striking similarity is that in both samples resilience was strongly related to high Self-directedness, high Persistence, and low Harm Avoidance. The most striking difference was that resilience was not correlated with Reward Dependence in the highly sociable family practitioners but was correlated with resilience in the less sociable university students studying natural science or liberal arts degrees. These findings suggest that resilience is influenced by multiple personality components that may differ between populations varying in levels of adversity and cultural context.

More generally, in our sample of predominantly Caucasian physicians, Reward Dependence was weakly correlated with Self-directedness, strongly correlated with Cooperativeness, but not with Resilience. Additionally the study of Korean students specifically compared males and females in both levels of TCI traits and their relationships with measures of Resilience. Most surprising was that their sample of men was higher in Cooperativeness, whereas Caucasian women are usually much higher in Cooperativeness than Caucasian men (Cloninger et al., 1994; Eley, Young & Przybeck, 2009a; Eley, Young & Prysbeck, 2009b). We found that only Harm Avoidance was significantly higher in females, which is congruent with previous studies of Caucasians that show females as higher in Harm Avoidance, Reward Dependence and Cooperativeness compared to males (Parker, Cheah & Parker, 2003; Eley, Young & Przybeck, 2009a; Eley, Young & Prysbeck, 2009b). These findings are likely explained by cultural differences as noted by Kim and others (Kim, Lee & Lee, 2013; Al-Halabi et al., 2011).

Our study specifically focussed on the relationship between a single measure of resilience and personality trait levels of family medicine doctors, a single professional group which regularly works in an environment with significant stress and pressure. However this does not preclude other environmental influences from impacting on a doctor’s resilience. Environmental factors influence the development of personality because stress and demoralization can increase Harm Avoidance (Svrakic, Przybeck & Cloninger, 1992) whereas safe and supportive environments allow healthy maturation of character traits like Self-directedness and Cooperativeness (Cloninger, 2004; Josefsson et al., 2013). Accordingly, the health care environment is likely to be important as an influence on resilience and well-being in medical students and doctors. Whatever promotes the personal well-being of physicians is likely to enhance their ability and longevity as effective health-care providers in the health care system. The further utility of the close relationship between resilience, well-being and personality may have implications as an adjunct to selection processes, as well as in health promotion and treatment efforts among medical students and physicians.

**Figure 1:**
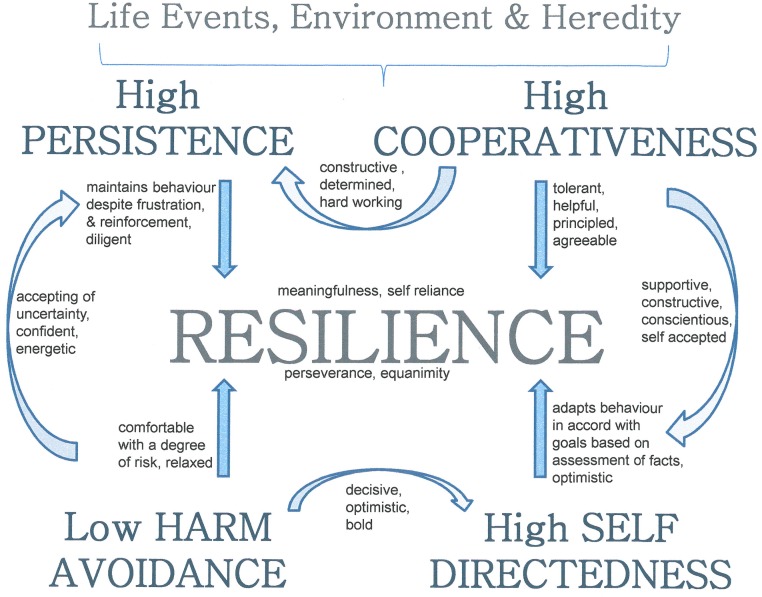
The relationship between temperament and character traits and resilience. Description of the relationships between key temperament and character traits and resilience. All relationships have a significant Pearson product-moment correlation coefficient at the 0.001 level (2-tailed). Strength of correlations are medium: *r* = .30 to .49 and strong: *r* = .50 to 1.0 (see Table 3 for detail).

Selection of students into medical school remains highly contentious and various models include the use of standardised non-cognitive tests to try and identify desirable or undesirable traits (Bore & Munro, 2009; Powis, 2009). It could be argued that medical school selection should focus on finding a broad cohort of capable and stable students with a positive attitude toward their medical career. Students who will cope with their workload, maintain their curiosity and commitment, and have an open mind to try new things, accept failure, learn from it and move on. These attributes are the fundamental basics of resilience and well-being as they are applicable to stable successful doctors. Clearly we should seek medical students who are resilient and mature, as described here, while recognizing that people can be helped to develop in resilience and well-being (Cloninger & Cloninger, 2011). We have shown that high resilience is associated with a mature and stable personality profile consistent with these attributes and now summarize them and their relevance to doctors as illustrated in Fig. 1.

The positive associations between measures of Resilience and character traits of Self-directedness and Cooperativeness indicate mature characteristics for doctors. Self-directedness reflects a subject-object dichotomy of self-concept (i.e., responsible versus blaming) and quantifies the extent to which an individual is responsible, reliable, resourceful, goal-oriented and self-confident. Individuals who are self directed accept responsibility for problems that occur so they can move on and learn from their mistakes. Cooperativeness reflects the concept of one’s connection with others (i.e., empathic versus insensitive) and quantifies the extent to which individuals are tolerant, helpful, forgiving, and principled. Persons who are highly cooperative and understanding are able to accept and empathise with others’ opinions or behaviours, even if contrary to their own. They don’t lose sight of their own principles but work out a solution to achieve the best outcome for everyone (Cloninger & Zohar, 2011). These positive relationships imply a positive affective style which is highly desirable in doctors and health professionals in general.

Resilience has a strong negative correlation with the temperament trait Harm Avoidance which reflects a heritable bias observed as anxiety and pessimistic worry in anticipation of problems. This inverse relationship suggests that persons low in Harm Avoidance are less anxious, more decisive and can confidently communicate with colleagues and patients. These individuals are optimistic, comfortable with accepting a degree of risk and are better at adapting to challenge than individuals who are negative and worry (Simeon et al., 2007). However this does not exclusively imply that someone who is anxious or cautious cannot be resilient. Certainly anxious people are able to cope well with adversity depending on their levels of the character traits; Self-directedness and Cooperativeness. Because every individual has a certain level of every trait – it is the combination of the various levels of these traits that builds everyone’s unique personality profile (Cloninger, 2004; Campbell-Sills, Cohan & Stein, 2005).

The temperament trait Persistence plays a particularly pivotal role in a resilient personality because it indicates a heritable bias of maintaining behaviour despite frustration, fatigue or other difficulties (Cloninger et al., 2012). The role of high Persistence in resilience supports the notion that individuals who are determined and persevering will bounce back from setbacks or adversity. Our findings that high Persistence, low Harm Avoidance, and high Self-directedness contribute strongly to resilience confirms the importance of this synergistic profile in health promotion (Cloninger et al., 2012). However, while the close relationship between Resilience and high Persistence appears outwardly desirable, there is a caveat to high Persistence – the costs of perfectionism. Perfectionism is common in doctors, medical students and high achieving individuals but is inevitably self defeating and can lead to burn-out with increased levels of anxiety when a person struggles to do what may be impossible (Cloninger et al., 2012).

The literature is clear about the importance of having high levels of resilience in stressful, harsh or uncertain environments – all of which are common in medical practice (Howe, Smajdor & Stockl, 2005; Haglund et al., 2009; Drybye & Shanafelt, 2012). The strong significant associations between resilience and certain personality traits are in agreement with literature on their association with well being (Schmutte & Ryff, 1997; Ryan & Deci, 2000; Cloninger, 2004; Cloninger & Zohar, 2011) which is primarily related to high Self-directedness, Cooperativeness and Persistence. Only Harm Avoidance is not as strongly associated with well being (Keyes, Shmotkin & Ryff, 2008; Cloninger & Zohar, 2011) and is congruent with our finding of its negative correlation with resilience and every other trait.

Psychological maturity is relevant to understanding individuals who choose to undertake challenging work because people such as doctors who regard their work experiences as meaningful and purposeful are better able to cope and make the most of any life circumstance. In this regard, Self-directedness and Persistence stand out as consistent predictors of a mature personality (Cloninger & Zohar, 2011).

The associations between Resilience, Self-directedness and Persistence further implies that resilience should be considered as part of a profile that promotes well being and the ability to cope in medical training and practice. While the way to build resilience is to deal with life challenges as they arise, it is useful to cultivate a mature and adaptable personality in order to be well equipped to cope with and bounce back from life’s challenges (Richardson, 2002). It is important to understand that personality traits are not fixed; rather resilient personality traits can be developed. Increasing self-awareness of one’s personality leads to an understanding of their strengths and weakness in adapting to life’s challenges, and predict an individual’s negative and positive aspects of well being (Cloninger & Cloninger, 2011). This has implications for selection and counselling into medical school and is noted as an area that requires more research (Hodgson et al., 2007; Powis, 2008). Perhaps more important is its application for the provision of educational programs to support and improve the well being of doctors and students (Krasner et al., 2009).

### Limitations

The study has several limitations which limit the generalisabilty of our findings. The sample population is a specific group and may not represent the general population. We have no information on individual participant levels of depression, anxiety, physical or mental illness which may influence our results, but the effects of current mood on personality are generally weak (Svrakic, Przybeck & Cloninger, 1992; Cloninger, 2004). The data is cross-sectional, which prohibits any causal conclusions from these findings alone, but other longitudinal studies of predictive validity support our interpretations (Gil & Caspi, 2006; Grucza & Goldberg, 2007; Josefsson et al., 2011; Cloninger et al., 2012; North & Cloninger, 2012; North, Abbacchi & Cloninger, 2012). It is self-reported and self-selected which may introduce bias from participants with a greater interest in the nature of this research. Additionally we do not have data on non-responders. The sample comprised only FP across Australia and sampling other disciplines including students will improve the validity of our data. The sample size is modest but a 61% response rate in busy FPs is encouraging.

## Conclusions

The concept of resilience has much utility for training and professional development in medical students and doctors. The inclusion of resilience alongside research on personality trait patterns provides an adjunct to enhancing the counselling of medical students and doctors through an increased understanding of what traits are most associated with their well being. The key personality traits which are conducive to enhance or impair resilience can be developed and in turn nurture a more resilient personality better equipped to adapt to the stressors of medicine. Further research is underway which explores resilience and personality across other professional groups.
